# Natural Killer Cell Dysfunction in Premenopausal *BRCA1* Mutation Carriers: A Potential Mechanism for Ovarian Carcinogenesis

**DOI:** 10.3390/cancers16061186

**Published:** 2024-03-18

**Authors:** Shaun Haran, Kantaraja Chindera, May Sabry, Nafisa Wilkinson, Rupali Arora, Agnieszka Zubiak, Thomas E. Bartlett, Iona Evans, Allison Jones, Daniel Reisel, Chiara Herzog, Twana Alkasalias, Mark Newman, Jaeyeon Kim, Angelique Flöter Rådestad, Kristina Gemzell-Danielsson, Adam N. Rosenthal, Louis Dubeau, Mark W. Lowdell, Martin Widschwendter

**Affiliations:** 1 Department of Women’s Cancer, Elizabeth Garrett Anderson (EGA) Institute for Women’s Health, University College London (UCL), 72 Huntley Street, London WC1E 6DD, UK; s.haran@ucl.ac.uk (S.H.); k.chindera@ucl.ac.uk (K.C.); iona.evans@ucl.ac.uk (I.E.); allison.jones@ucl.ac.uk (A.J.); d.reisel@ucl.ac.uk (D.R.); adam.rosenthal@ucl.ac.uk (A.N.R.); 2 Department of Gynaeoncology, University College London Hospitals (UCLH) NHS Trust, London NW1 2BU, UK; 3 Institute of Immunity & Transplantation, Royal Free Hospital, Rowland Hill Street, London NW3 2PF, UK; may.sabry.10@alumni.ucl.ac.uk (M.S.); a.zubiak@ucl.ac.uk (A.Z.); m.lowdell@ucl.ac.uk (M.W.L.); 4 Department of Cellular Pathology, University College London Hospitals (UCLH) NHS Trust, London NW1 2BU, UK; nafisa.wilkinson@nhs.net (N.W.); rupali.arora@ucl.ac.uk (R.A.); 5 Department of Statistical Science, University College London, London WC1E 7HB, UK; thomas.bartlett.10@ucl.ac.uk; 6 European Translational Oncology Prevention and Screening (EUTOPS) Institute, University of Innsbruck, 6060 Hall in Tirol, Austria; chiara.herzog@uibk.ac.at; 7 Research Institute for Biomedical Aging Research, University of Innsbruck, 6020 Innsbruck, Austria; 8 Department of Women’s and Children’s Health, Division of Obstetrics and Gynecology, Karolinska Institutet, Karolinska University Hospital, 14186 Stockholm, Sweden; twana.alkasalias@ki.se (T.A.); angelique.floter-radestad@ki.se (A.F.R.); kristina.gemzell@ki.se (K.G.-D.); 9 General Directorate of Scientific Research Center, Salahaddin University-Erbil, Erbil 44002, Iraq; 10 Precision Analytical Inc., McMinnville, OR 97128, USA; mnewman@dutchtest.com; 11 Department of Biochemistry and Molecular Biology, Indiana University Melvin and Bren Simon Comprehensive Cancer Center, School of Medicine, Indiana University, Indianapolis, IN 46202, USA; jaeyeonk@iu.edu; 12 Department of Pathology, Keck School of Medicine, USC Norris Comprehensive Cancer Centre, University of Southern California, Los Angeles, CA 90033, USA; ldubeau@usc.edu

**Keywords:** *BRCA1*, natural killer cells, immune surveillance, epithelial ovarian cancer

## Abstract

**Simple Summary:**

Since the discovery of a *BRCA1* mutation nearly 30 years ago, definitive methods for ovarian cancer-risk reduction has remained unchanged, requiring carriers of a pathogenic mutation to consider undergoing surgical removal of both fallopian tubes and ovaries from the ages of 35 to 40 years. The sequelae of these invasive measures include a profound impact on general health, psychological well-being, sexual and reproductive function. Identifying targetable aberrances in *BRCA1* mutation carriers, such as sex hormones, or adopting mechanisms aimed at enhancing tumor immunosurveillance may facilitate the reduction of early cancer-promoting events. This is particularly relevant for the fimbrial fallopian tube, where the majority of epithelial ovarian cancers originate. Utilizing a proof-of-concept for potentially targetable aberrances may further the development of non-surgical adjuncts or surveillance methods aimed at cancer-risk reduction for women deferring or declining surgery.

**Abstract:**

Background: Tissue-specificity for fimbrial fallopian tube ovarian carcinogenesis remains largely unknown in *BRCA1* mutation carriers. We aimed to assess the cell autonomous and cell-nonautonomous implications of a germline *BRCA1* mutation in the context of cancer immunosurveillance of CD3^−^ CD56^+^ natural killer (NK) cells. Methods: Premenopausal *BRCA1* mutation carriers versus age-matched non-carriers were compared. Daily urinary 5β-pregnanediol levels were used to determine progesterone metabolomics across an ovarian cycle. Using peripherally acquired NK cells the cell-mediated cytotoxicity of tumor targets (OVCAR-3, K-562) was determined using live cellular impedance (xCELLigence^®^) and multicolor flow cytometry. Hypoxia-inducible factor 1-alpha (HIF-1α) immunohistochemistry of cancer-free fallopian tube specimens allowed a comparison of proximal versus distal portions. Utilizing these findings the role of environmental factors relevant to the fimbrial fallopian tube (progesterone, hypoxia) on NK cell functional activity were studied in an ovarian phase-specific manner. Results: *BRCA1* mutation carriers demonstrate a differential progesterone metabolome with a phase-specific reduction of peripheral NK cell functional activity. Progesterone exposure further impairs NK cell-mediated cytotoxicity in a dose-dependent manner, which is reversed with the addition of mifepristone (1.25 µM). The fimbrial fallopian tube demonstrated significantly higher HIF-1α staining, particularly in *BRCA1* mutation carriers, reflecting a site-specific ‘hypoxic niche’. Exposure to hypoxic conditions (1% O_2_) can further impair tumor cytotoxicity in high-risk carriers. Conclusions: Phase-specific differential NK cell activity in *BRCA1* mutation carriers, either systemically or locally, may favor site-specific pre-invasive carcinogenesis. These cumulative effects across a reproductive lifecycle in high-risk carriers can have a detrimental effect further supporting epidemiological evidence for ovulation inhibition.

## 1. Introduction

The inheritance of a pathogenic germline *BRCA1* mutation predisposes individuals to a 65.6% (95% CI, 12.8–86.4%) lifetime risk of ovarian carcinogenesis, with the most prevalent histological subtype arising from the fimbrial fallopian tube [1]. Despite the ubiquitous expression of BRCA1, explanations for its site-specificity appear to go beyond dysfunctional deoxyribonucleic acid (DNA) repair alone [2]. Natural killer (NK) cells represent a vital component of tumor immunosurveillance, which is fundamental for host protection and survival [3]. These circulating lymphocytes demonstrate an ability for potent cell-mediated cytotoxicity without prior sensitization, unlike T-cells, and homing abilities that allow them to infiltrate inflammatory niches via chemotactic migration. NK cell cytotoxic activity is inversely associated with cancer incidence and utilizing peripherally acquired NK cells may serve as a prognostic marker [4,5]. For both tissue-resident and circulating NK cells consideration is needed for the microenvironment of interest given that differential immune cell activity is observed within the gynecological tract [6]. Challenges remain with the current efficacy of immune checkpoint inhibitors for high-grade serous ovarian cancers (HGSOC), due to the heterogeneity of this disease process and variations in individual immunological responses [7]. This emphasizes the need to identify site-specific cell-extrinsic factors modulating a microenvironment, particularly when deciphering phenotypic divergences in anti-tumour activity [8].

The fimbrial fallopian tube is in close vicinity to the steroid hormone-producing ovary resulting in a local niche vulnerable to the cyclical exposure of highly inflammatory follicular fluid during ovulation. Ovulatory events, occurring ~day 14 of a 28-day menstrual cycle, result in the phase-specific fluctuation of sex hormones that are attributable to immunoregulatory differences and sexual dimorphism in premenopausal women [9]. Progesterone, as the predominate component of follicular fluid, is also released locally by the corpus luteum (~40 mg per day) resulting in a unique tissue gradient not observed in peripheral blood or elsewhere in the body. Unlike estrogen, which rises in the mid-follicular phase followed by a mid-luteal secondary peak, progesterone secretion is phase-specific, with levels up to 250-fold higher in the luteal phase than in the follicular phase [10]. In *BRCA1/2* mutation carriers luteal phase progesterone levels are significantly higher than non-carriers, with evidence supporting physiological dysregulation in germline carriers as a potential tumorigenic mechanism [11,12,13]. The use of anti-progestins significantly reduces the metastatic potential of HGSOC further favoring progesterone as an immunosuppressant with a functionally relevant role in *BRCA1* mutation carriers [14]. Other site-specific features unique to the fimbrial microenvironment include a susceptibility to a reduced oxygen gradient or tissue hypoxia, given that this blind-ending site is distal to the highly vascular uterine cornua. Due to technical complexities and ethical issues accessing viable NK cells from this site, without complete surgical excision, the study of peripheral blood NK cells offers a useful surrogate marker for assessing baseline anti-tumor functionality.

Our objective was to determine whether NK cell cytotoxic activity is (i) differential due to the cell-autonomous presence of a germline *BRCA1* mutation, and/or (ii) aberrant in response to cell-nonautonomous effects (i.e., supraphysiological progesterone and hypoxia) with consideration of the fimbrial fallopian tube.

## 2. Materials and Methods

All subjects gave their informed consent for inclusion before they participated in the study. The study was conducted in accordance with the Declaration of Helsinki and the protocol was approved by the appropriate Institutional Review Board (IRB) in accordance with the local research ethics committee (REC).

### 2.1. Subjects

All samples were derived from women in Central London recruitment sites: (1) aged 18- to 45-years; (2) in good general health (including body mass index <25); (3) with regular menstrual cycles (25- to 35-days); (4) no current or previous use of hormonal medication in the past 3 months (oral contraceptive pill, hormonal intrauterine device/Mirena^®^, depot injection, or contraceptive implant); (5) not currently pregnant or breastfeeding; (6) no previous diagnosis of cancer; (7) no prior surgical removal of both ovaries and/or fallopian tubes; (8) absence of concurrent infection. Where specified participants confirmed negative for *BRCA1/2* mutations (*BRCA1*/*2* wild-type; *BRCA1*/*2*wt) were compared to those confirmed positive for a *BRCA1* mutation (*BRCA1*mut).

### 2.2. Sample Collection

**Figure 1:**
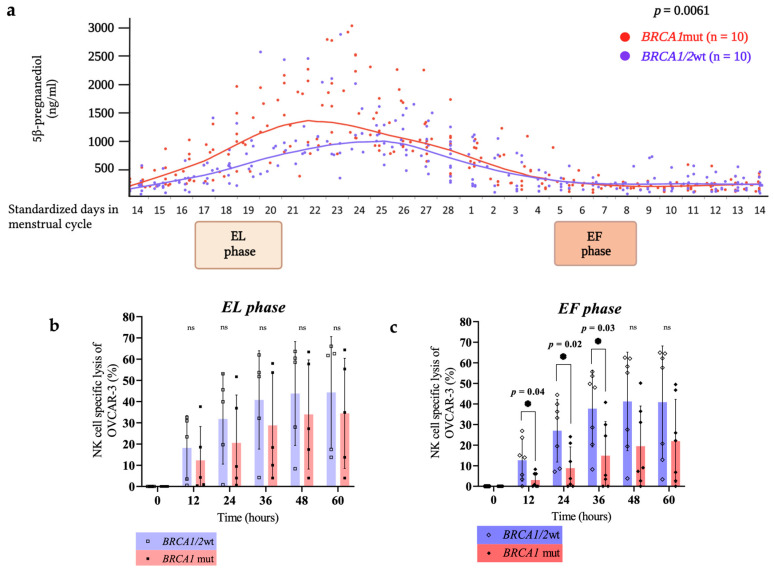

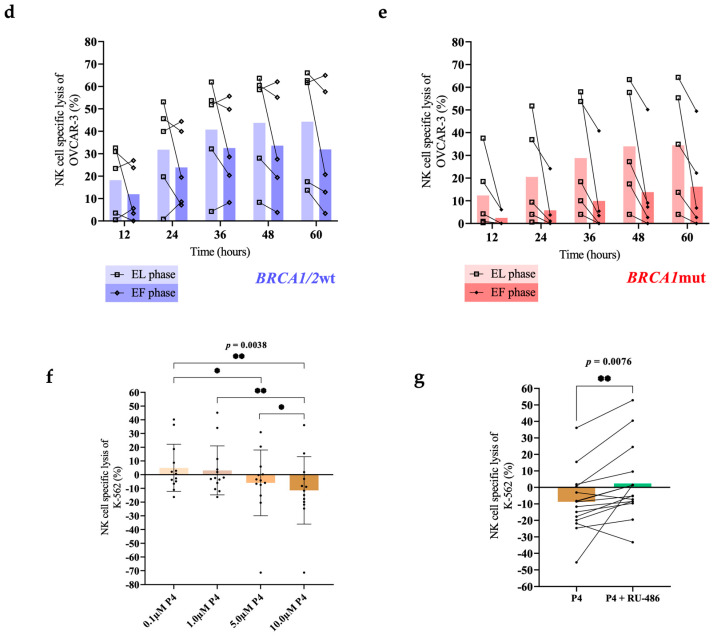
Ovarian cycle rhythmicity of progesterone dynamics and the impact on NK cell cytotoxic responses: (**a**) 5β-pregnanediol levels: Hormone profiles of progesterone metabolite 5β-pregnanediol are shown using daily urinary measurements to determine concentrations (ng/ml) in premenopausal *BRCA1* mutation carriers (*BRCA1*mut; 35.10 ± 5.23 years; *n* = 10) and women negative for *BRCA1/2* mutation (*BRCA1/2*wt; 34.33 ± 6.05 years; *n* = 10). Cell autonomous effects of a germline *BRCA1* mutation on peripheral blood NK cell functional activity was determined against OVCAR-3 using real-time cell impedance (60 h assay) (**b**–**e**) Utilizing a single menstrual cycle (standardized 28 calendar days) pre-defined sampling windows for the EL phase (3–6 days post ovulation) and EF phase (2–5 days post menstruation) in relation to ovulation (~day 14, confirmed using urine luteinizing hormone testing) were used to acquire NK cells. Cell-mediated cytotoxicity levels in the (**b**) EL phase (*n* = 5) and (**c**) EF phase (*n* = 7), were determined for *BRCA1/2*wt versus *BRCA1*mut. Mean values ± SD from technical replicates with data comparisons to determine those significant using two-tailed *t* tests (*p* < 0.05) and not significant (ns). Linked data points demonstrating NK cell-mediated cytotoxicity in the same participant are shown across different phases in (**d**) *BRCA1/2*wt and (**e**) *BRCA1*mut. To ascertain the direct ‘progesterone effect’ NK cells derived from healthy premenopausal participants in the EF phase were utilized against tumor targets (K-562) in flow cytometric studies (4 h assay) (**f**,**g**). The (**f**) ‘progesterone effect’ (*n* = 13) determined the dose-dependent effect of progesterone (P4) co-culture (0.1 µM to 10 µM) on NK cell cytotoxicity (mean ± SD). NK cells co-cultured with P4 overnight (day 0) were washed and re-suspended in fresh CM before NK cell-specific lysis of K-562 calculated (day 1). Using similar methods, the (**g**) ‘anti-progestin effect’ (*n* = 15) determined the effect of overnight co-culture in the highest concentration of P4 (10 µM) alone or with the addition of Mifepristone (RU486; 1.25 µM; day 0). Linked data is shown for the change in NK cell-mediated lysis of tumor target K-562 (day 1). Two-tailed *t* tests were used to determine statistical significance (*p* < 0.05; Supplementary Materials Table S2a–d).

All participants undertook menstrual calendar mapping and urinary luteinizing hormone testing (SureScreen Diagnostics Ltd., Nottingham, UK) for ovulation confirmation. During a single menstrual cycle (~28 days) daily collection of urine samples and timed collection of venous blood (individualized to each subject) was performed during the EL phase (days 3 to 6 days post-ovulation) and EF phase (days 2 to 5 days post cessation of menstruation)—the latter equating, for example, to days 5 to 8 of a regular menstrual cycle (Figure 1a).

### 2.3. 5β-Pregnanediol

Participants were provided with home testing kits (Dried Urine Testing for Comprehensive Hormones; DUTCH, Precision Analytical, McMinnville, OR, USA) that allowed the measurement of progesterone metabolites in the urine. Dried urine samples collected on filter paper were extracted, hydrolyzed, and derivatized prior to analysis (GC–MS/MS; 7890/7000B; Agilent Technologies, Santa Clara, CA, USA). Levels of 5β-pregnane-3α, 20α-diol (5β-pregnanediol, βPg) in premenopausal women were compared in *BRCA1/2*wt (*n* = 10) versus *BRCA1*mut (*n* = 10) (Figure 1a). Hormone concentrations were normalized to creatinine (assay sensitivity: βPg, 10 ng/mL) using a prior-validated assay (DUTCH, Precision Analytical, McMinnville, OR, USA).

### 2.4. Isolation of Peripheral Blood NK Cells

Fresh peripheral blood samples (Lithium Heparin Tubes, Becton Dickinson (BD) Vacutainer^®^ (Berkshire, UK) spray-coated with gel plasma separator) were collected between 8 a.m. and 1 p.m. at a designated sample collection site or research facility. Peripheral blood mononuclear cells (PBMCs) were isolated by Lymhoprep™ density gradient separation (Cedarlane^®^ Laboratories, Burlington, Canada) before −80 °C storage (Mr Frosty, Nalgene, Rochester, NY, USA) in cryoprotectant (DMSO; Sigma-Aldrich, St. Louis, MO, USA), followed by final storage (liquid nitrogen, −196 °C). NK cells were negatively selected (EasySep™ Human NK Cell Enrichment Kit; STEMCELL™ Technologies, Vancouver, BC, Canada; #19055) as per the manufacturer’s recommendations from PBMCs thawed in 20% FBS (viability > 85%), with the final enriched fraction assessed using flow cytometry (viability, purity, absolute cell count).

### 2.5. Cell Lines

Human cancer cell lines OVCAR-3 (ATCC^®^HTB-161^™^) and K-562 cells (ATCC^®^CCL-243^™^) were cultured as recommended by the repository. OVCAR-3 (high-grade serous ovarian adenocarcinoma cell line) cells were cultured in Gibco^™^ RPMI-1640 GlutaMAX™ supplemented with + 20% heat-inactivated fetal bovine serum (FBS), 100 U/mL penicillin, 0.1 mg/mL streptomycin (Invitrogen Ltd., San Diego, CA, USA), and 0.01 mg/mL insulin (Sigma-Aldrich). Adherent OVCAR-3 cells were harvested during the exponential growth phase using Detachin™ (Amsbio, Abingdon, UK) cell detachment solution. K-562 cells (chronic myelogenous leukemia cell line) were cultured in Gibco^™^ RPMI-1640 GlutaMAX™ (Gibco, Waltham, MA, USA), 10% FBS, 100 U/mL penicillin, and 0.1 mg/mL streptomycin. Suspension K-562 cells were harvested during the exponential growth phase.

### 2.6. Real-Time Cell Impedance-Based Cytotoxicity Assay

The xCELLigence^®^ RTCA (Real Time Cell Analyzer) MP (Multi-Plate) Instrument (Agilent Technologies, Inc., Santa Clara, CA, USA) was used to determine the cytotoxicity of adherent unlabeled target cells (OVCAR-3) in uncoated ultra-violet radiated polyethylene terephthalate (PET) E-Plates; 0.15 × 10^6^ OVCAR-3 cells were pre-seeded into microtiter wells to allow for cell attachment before the addition of unstimulated 0.75 × 10^6^ NK cells, using an effector:target (E:T) ratio of 5:1, once the cell index (CI) reached > 0.5. Live cellular impedance recordings were taken every 15 mins, under normoxic (21% O_2_) or hypoxic (1% O_2_) conditions where specified. NK cell cytotoxic responses that are ‘phase-specific’ are shown with samples derived from the EL phase: *BRCA1/2*wt (*n* = 5) versus *BRCA1*mut (*n* = 5; Figure 1b), or the EF phase: *BRCA1/2*wt (*n* = 5) versus *BRCA1*mut (*n* = 7; Figure 1c). To further describe a ‘phase-effect’ paired samples were used from the same individual to compare NK cell cytotoxicity in *BRCA1/2*wt (*n* = 5; Figure 1d) versus *BRCA1*mut (*n* = 5; Figure 1e), with groups age-matched <2-years.

### 2.7. Flow Cytometry-Based Cytotoxicity Assay

Target cells (K-562) recovered from suspension culture were washed in HBSS before resuspension in PKH-67-labeling diluent (Green Fluorescent Cell Linker Kit for General Cell Membrane Labelling; Sigma-Aldrich). The cell suspension was incubated for 3 min at room temperature in the dark and the labeling reaction was stopped by the addition of 1.0 mL FBS (1 min). Labeled cells, washed twice in Culture Medium (CM), were resuspended in CM at 10^6^/mL (viability > 85%). Phase-specific derived NK cells from healthy premenopausal participants (EF phase; *n* = 13) were co-cultured overnight (24 h) in CM alone or with progesterone (P4; Sigma-Aldrich) at different final concentrations (0.1 µM to 10 µM) before being washed, checked for viability, and added to K-562 the following day (Figure 1f). Then, 0.5 × 10^6^/mL NK cells were co-cultured (E:T, 5:1) with 0.1 × 10^6^/mL PKH-67-labeled targets (K-562) in RPMI-1640 GlutaMAX™ (10% FBS) for 4 h at 37 °C. Anti-progestin effects were also determined by co-culturing NK cells from healthy premenopausal participants (EF phase; *n* = 15) overnight in (i) media alone, with (ii) P4 (10 µM), or with (iii) P4 (10 µM) with Mifepristone (RU486; 1.25 µM; Sigma-Aldrich; Figure 1g); 1.25 µM Mifepristone has a ~25 mg oral equivalent [15]. TO-PRO-3 iodide (Invitrogen) was added (10% of total volume) to determine cytotoxicity (>10,000 events) using a NovoCyte^®^ flow cytometer (Agilent Technologies, Inc.). TO-PRO-3 iodide-positive cells and the background target cell death were calculated to determine phase-specific cell-mediated cytotoxicity, as measured by % cell lysis in experimental condition − % spontaneous lysis.

### 2.8. Immunohistochemistry

**Figure 2:**
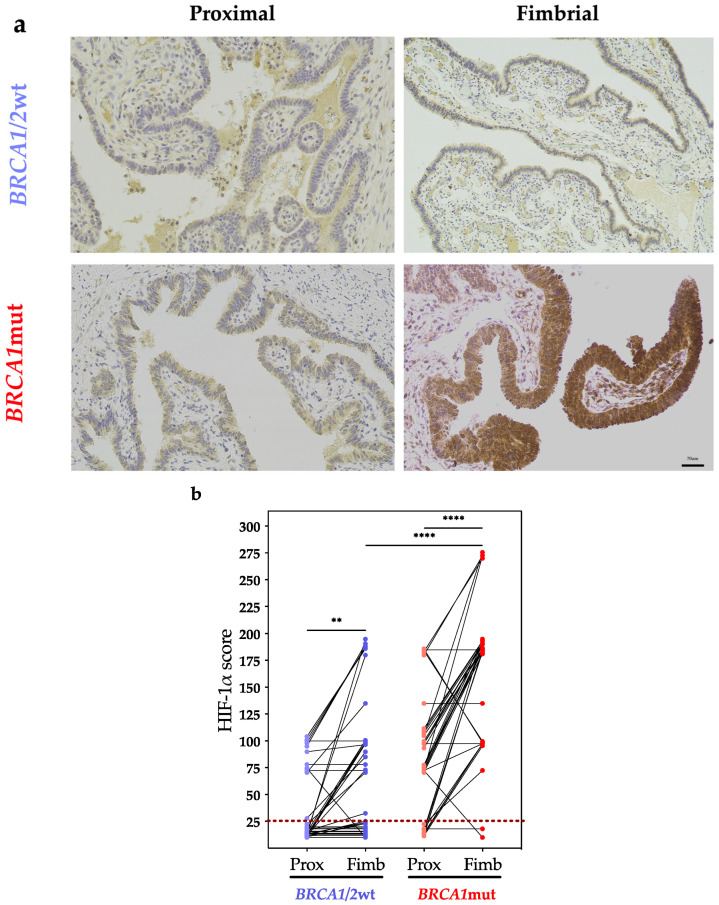

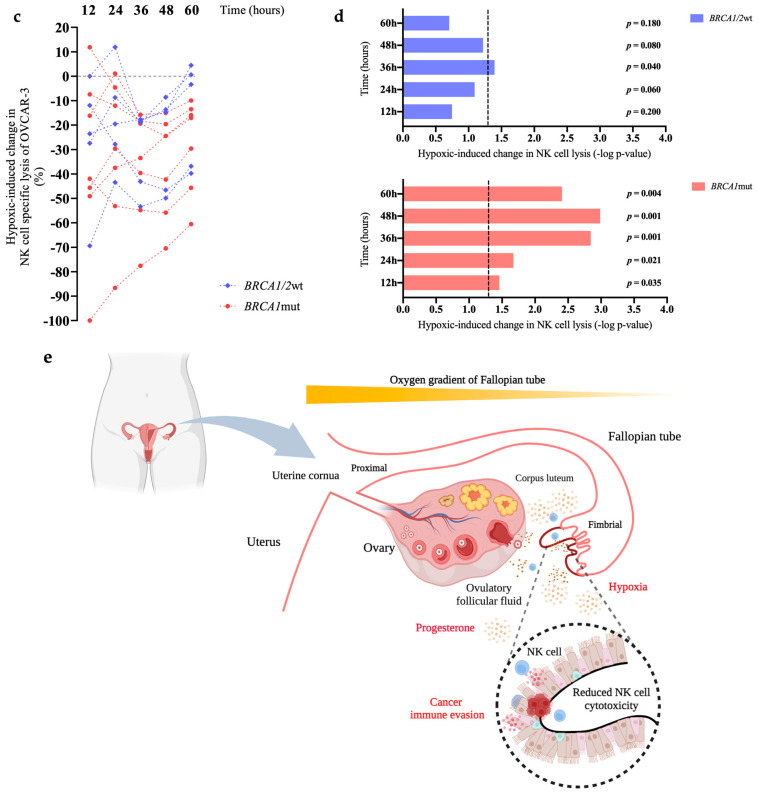
The role of hypoxia in the fimbrial fallopian tube and its impact on NK cell cytotoxic responses: (**a**) Cytoplasmic HIF-1α immunohistochemistry in paired fallopian tube ends in *BRCA1/2*wt vs. *BRCA1*mut (scale bar: 70 μm). (**b**) HIF-1α scores (percentage of cells showing positive staining multiplied by intensity) of proximal (Prox) versus fimbrial (Fimb) fallopian tubes in the same subject: *BRCA1/2*wt (*n* = 37; ** *p* < 0.001), *BRCA1*mut (*n* = 37; **** *p* < 0.0001), paired *t* test. For visualization purposes, samples with the same HIF-1α score (Supplementary Materials Figure S1) are stacked and those samples below the dotted line considered negative for staining. (**c**) Percentage change in OVCAR-3 NK cell-specific cytotoxicity in hypoxia (1% O_2_) versus normoxia (21% O_2_) for the EF phase: *BRCA1/2*wt (*n* = 5), *BRCA1*mut (*n* = 7). (**d**) Log transformed *p*-values for data shown in (**c**). Bars right of the dotted line are significant (*p* < 0.05; Supplementary Materials Table S2f). (**e**) Proposed mechanism for cancer immuno-evasion at the fimbrial fallopian tube. Image created with Biorender.com (free trial; accessed 27 January 2024).

Hypoxia-inducible factor (HIF)-1α staining of fimbrial and proximal paired-ends of human fallopian tubes, free of serous tubal intraepithelial carcinoma and cancer, was performed (*n* = 37) using 3 mm sections from formalin-fixed paraffin-embedded (FFPE) tissue blocks (UCL Biobank, REC reference: 14/LO/1633, NC09.13; Figure 2a). Staining (Leica Bond III automated staining platform) used Bond Polymer Refine detection (Leica, DS9800) with 3,3′-Diaminobenzidine (DAB) chromogen. Heat-induced epitope retrieval was performed for 20 min at 99 °C (Leica, Wetzlar, Germany; AR9640) and peroxide blocking for 5 min. The primary antibody (HIF-1α, Novus Biologicals, mouse monoclonal H1alpha67, cat. no. NB100-105) was diluted 1:100 in Leica Bond Primary Antibody Diluent (60 min). Rabbit anti-mouse post-primary reagent was applied as per the kit’s recommendations, which were enhancement with 0.5% copper sulphate for 5 min followed by a Hematoxylin counterstain (45 s). HIF-1α staining intensity was independently reviewed by two pathologists blinded for *BRCA* status. HIF-1α positive cells (0–100) and average staining intensity (0, negative to +++, highest positivity) generated “HIF-1α scores”, derived by multiplying the number of positive cells and the respective staining intensity (Figure 2b). Cytoplasmic HIF-1α values for each sample are shown in the Supplementary Materials (Table S1). The slides were scanned using a NanoZoomer digital scanner.

### 2.9. Data Analysis

Cellular impedance was utilized to determine the % cell lysis of tumor targets over 60 h (37 °C) using xIMT software (Version 2.3.2) to generate mean values of experimental replicates. Representative example plots using for flow cytometry is shown in the Supplementary Materials (Figure S1a–c). Quantitative HIF-1α staining was analyzed using NDP.view2 software (Version 2.0, Hamamatsu Photonics, Welwyn Garden City, UK) and generated HIF-1α scores shown (Table S1). Using a two-tailed Student *t* test (GraphPad Prism, Version 9.5.0); *p* < 0.05 is considered statistically significant, with absolute *p* values provided (Table S2a–f).

## 3. Results

Firstly, we assessed whether the dynamics of progesterone metabolomics were differential based on germline *BRCA1* mutational status. Using daily first-catch dried urine spots, progesterone metabolite (5β-pregnanediol) concentrations were directly compared between *BRCA1* mutation carriers (*n* = 10) and non-carriers (*n* = 10) across one entire menstrual cycle (Figure 1a). To standardize menstrual cycles between participants levels of 5β-pregnanediol are shown from the first day of confirmed ovulation (corresponding to day 14 of a standardized menstrual cycle). We identified 5β-pregnanediol concentrations to be significantly higher overall in *BRCA1*mut (*p* = 0.0061), which also demonstrated an observation of ovarian-phase specificity with luteal dominance.

To determine further ovulatory effects on NK cell cytotoxicity, we compared real-time cell-mediated tumor lysis in the EL and EF phases of a menstrual cycle against HGSOC targets (NIH:OVCAR-3; *ATCC* HTB-161^™^) using live cellular impedance (xCELLigence^®^; Agilent; 60 h assay). Absolute mean levels of cytotoxicity were reduced in *BRCA1*mut (*n* = 5) relative to *BRCA1/2*wt (*n* = 5) but did not reach significance in the EL phase (*p* = 0.392; Figure 1b), whereas significantly lower NK cell cytotoxicity was noted in the EF phase (*p* < 0.05; Figure 1c) in *BRCA1*mut (*n* = 7) at 12, 24, and 36 h relative to non-carriers (*n* = 5). When using paired samples from the same individual phase-specific activity was inconsistent in *BRCA1/2*wt (*n* = 5; Figure 1d). However, in all *BRCA1*mut there was consistently lower EF phase cytotoxicity relative to the EL phase (*n* = 5; Figure 1e).

The potential for *BRCA*-specific immunomodulation in the EF phase may reflect the difference in progesterone exposure in vivo between premenopausal women, given that the EF phase follows a period of luteal dominant exposure to both progesterone and its metabolite 5β-pregnanediol in carriers [12]. To determine the implications of direct progesterone exposure, which may be detrimental during a theoretical window period of dysregulated NK cell activity, NK cells were co-cultured with progesterone in a dose-dependent manner (0.1 to 10 µM). OVCAR-3 has been shown to demonstrate progesterone receptor expression and is capable of cellular inhibition by Mifepristone which may have implications for cytotoxic assays assessing progesterone co-culture; therefore, an independent tumor target widely used in NK cell biology (K-562) was preferentially selected using an alternative cytotoxic assay (NovoCyte^®^). Overnight co-culture with progesterone (24 h) induced a highly significant reduction in NK cell cytotoxicity of tumor targets at differing concentrations (10 µM vs. 0.1 µM progesterone; *n* = 13; *p* = 0.0038; Figure 1f). At lower concentrations (0.1 µM; ~31 ng/mL), progesterone-induced effects were variable—even immunostimulating. Crucially, the co-culture of Mifepristone (1.25 µM; ~25 mg oral equivalent) significantly reversed the inhibition of NK cell cytotoxicity even at the highest progesterone concentration (10 µM) in matched donor samples (*n* = 15; *p* = 0.0076; Figure 1g).

Homing in on the fallopian tube microenvironment, a comparison of paired proximal versus fimbrial ends demonstrated the cytoplasmic immunoreactivity of HIF-1α (Figure 2a) to be higher at the fimbrial portion (Figure 2b). In *BRCA1*mut (*n* = 37) this site was significantly more ‘hypoxic’, with greater HIF-1α scores relative to *BRCA1/2*wt (*n* = 37; *p* < 0.001). The functional implications of hypoxia on anti-tumor responses relevant to HGSOC predisposition were determined using EF phase NK cells in parallel cytotoxic assays (xCELLigence^®^; 60 h) under normoxic (21% O_2_) versus hypoxic (1% O_2_) conditions, specifically against HGSOC target OVCAR-3. Both acute (<12 h) and sustained exposure to hypoxia (up to 60 h) significantly reduced NK cell cytotoxicity in *BRCA1*mut (*n* = 7) relative to age-matched non-carriers (*n* = 5; Figure 2c,d). This ‘hypoxic-induced’ effect was consistent across all time points and maximal at 48 h in germline carriers (*p* = 0.001; Figure 2d).

## 4. Discussion

We are the first to report on urine 5β-pregnanediol levels, HIF-1α expression in the fallopian tube, and peripheral blood NK cell activity in premenopausal women significantly predisposed to a higher lifetime risk of ovarian carcinogenesis. NK cell activity is shown with specific reference to (i) an ovarian cycle phase, exposure to (ii) supraphysiological progesterone, and (iii) environmental hypoxia. Collectively, these factors contribute to dynamics within the tissue and immune microenvironment of the fimbrial fallopian tube. Given that our findings were identified from otherwise ‘healthy’ premenopausal women, including those not taking hormonal contraception, they highlight the potential scope for altered tumor immunosurveillance within progesterone-dense or hypoxic niches.

Urinary progesterone metabolites provide a readily accessible, non-invasive, biological surrogate for blood samples that can mimic serum progesterone curves—also aligning with previously published findings [12,13]. The implications of sustained progesterone exposure or the direct effect on NK cells is not well understood. Inconsistencies in the literature can be attributable to older publication dates, limited reports, assay modalities, varying progesterone concentrations, male sex inclusion, and lack of ovarian cycle phase consideration when under-taking initial biological sampling [16,17,18,19]. Progesterone exposure can induce NK cell apoptosis and has been shown to alter activity in a concentration-dependent manner [18,19]. Further functional importance should be placed on local concentrations (ipsilateral to a corpus luteum or within the adnexa), given peritoneal levels can be 63-fold higher relative to serum and remain high ~7-days post-ovulation [10]. Specifically in *BRCA* mutation carriers circulating progesterone levels can be up to ~121% higher, further compounding these effects and supporting the need to minimize sequential ovulatory cycles as part of ovarian cancer chemoprevention [12,20].

Hypoxia is a core component of an evolving tumor microenvironment that has been shown to inhibit NK cell activity [2]. Real-time determination of in vivo fallopian tube oxygenation is challenging, emphasizing the need for the use of HIF-1α as a reliable proxy [21]. Concerningly for carriers higher fimbrial HIF-1α may also reflect synergy with *BRCA1* loss as a potential cancer-driving mechanism [22]. These effects may induce ‘hallmark qualities’ that are fundamental to cellular pathogenesis, immune evasion, and an evolving tumor microenvironment [2]. NK cell plasticity and immune contexture is heavily influenced by the interaction of mediators that modulate the functional and transcriptomic profile, as seen following cytokine- or tumor-priming [23]. Utilizing unstimulated NK cells minimizes bias when describing a baseline functional profile in healthy women (devoid of a clinically confirmed cancer), demonstrating superiority over *BRCA1*-deleted murine models.

Whilst aberrant differences pertaining to a germline *BRCA1* mutation are observed, we appreciate that the study is limited by its small size, emphasizing future scope to consider larger numbers or other target cell lines. A selection bias favored premenopausal women not using hormonal contraception; however, this was to minimize potential interference when determining a direct progesterone-mediated effect. Utilizing cryopreserved PBMCs can further affect the availability of viable NK cell frequencies which has implications when performing multiple cell-based assays from individual donors. Whilst differences in absolute cytotoxic values can be observed in the literature, this is dependent on experimental design and modality chosen. Importantly, a transient reduction in NK cell functional activity can have cancer incidence implications [4,5]. Cumulative exposure to cyclical periods of relatively lower NK cell tumor cytotoxicity, across the reproductive lifecycle of a *BRCA1* mutation carrier, may in turn have functional relevance from a cancer predisposition perspective.

There is a need to shift the paradigm towards cancer prevention, which requires a better understanding of precancerous disease states. In HGSOC, phylogenetic prediction of early disease evolution can be limited by its vast genetic heterogeneity. Site-specific differences within the adnexa have demonstrated scope for understanding potential mechanisms for tumor immune evasion [8]. Cyclical or sustained impairment of NK cell immune surveillance may be further potentiated by factors specific to the fimbrial niche, which is of functional importance for these high-risk individuals (Figure 2e). Oral contraceptive agents have already demonstrated an evidence-based benefit; however, patient-centered counselling is needed—particularly when using progestin-only agents during early reproductive years [24]. Larger feasibility studies should consider anti-progestin use or Mifepristone for its potential role in *BRCA1* mutation carriers, as part of individualized chemoprevention, given its beneficial experimental effects in HGSOC and cancer field defects in normal human breast tissue [13,14]. Whilst the use of aspirin may demonstrate benefits for solid tumors or for those with hereditary cancer predisposition (i.e., Lynch syndrome), its efficacious role in ovarian cancer requires further exploration alongside targeted agents aimed at HIF-1α or improving tissue oxygenation [25]. Since the identification of *BRCA1* nearly 30 years ago unfortunately little has changed for definitive ovarian cancer risk-reduction. Younger carriers now consider undergoing invasive risk-reducing surgery (bilateral salpingo-oopherectomy), which itself results in significant morbidity due to a surgical menopause which induces physiological effects, psychological burden, and loss of fertility [26]. Given the earlier age of ovarian cancer onset linked with a germline *BRCA1* mutation, efforts to minimize cancer-initiating events are needed particularly in the context for those declining or deferring surgery.

## 5. Conclusions

NK cells represent a major component of anti-tumour responses and understanding mechanisms that alter the cytotoxic potential within sites susceptible to carcinogenesis, such as the fimbrial fallopian tube, are imperative for women significantly predisposed to ovarian cancer. We propose that a precision-based individualized approach is adopted to target aberrantly released factors in *BRCA1* mutation carriers to improve peripheral NK cell activity. Mechanisms aimed at enhancing NK cell immunosurveillance specifically within the fimbrial fallopian tube can potentially narrow a carcinogenic ‘window period’, thus preventing cancer immune escape.

## Data Availability

The data presented in this study are available on request from the corresponding author.

## Supplementary Materials

The following supporting information can be downloaded at: https://www.mdpi.com/article/10.3390/cancers16061186/s1, Table S1: Quantitative representation of cytoplasmic HIF-1α protein levels in the proximal and fimbrial end of the Fallopian Tube; Table S2: Statistical Analysis; Figure S1: Representative plots.
