# Supplementary material for: Natural Killer Cell Dysfunction in Premenopausal BRCA1 Mutation Carriers: A Potential Mechanism for Ovarian Carcinogenesis
Source: Cancers (Basel). 2024 Mar 18;16(6):1186. doi: 10.3390/cancers16061186 (PMC10968968; doi:10.3390/cancers16061186)
Supplement: Supplementary file 1 [file cancers-16-01186-s001.zip › Supplementary Tables.pdf]

cancers MDPI

Table S1: Quantitative representation of cytoplasmic HIF-1α protein levels in the proximal and fimbrial end of the Fallopian Tube. The HIF-1α cytoplasmic intensity is graded from 0 (negative/weak) to 3+ (dense staining). The % of cells showing cytoplasmic positivity are counted. The average of % positive cells and intensity recordings from two pathologists (NW and RA) are shown. HIF-1α score = % of cells showing positive staining x intensity.

Tube number	Mutation	age	% of positive cells at the fimbrial end	% of positive cells at the proximal end	Intensity at fimbrial end	Intensity at proximal end	Fimbrial HIF1-1α Score	Proximal HIF-1α score	HIF-1α gradient
1	*BRCA1*	42	87.50	0	1	0	87.5	0	Fimbrial
2	*BRCA1*	42	62.5	12.5	1	1	62.5	12.5	Fimbrial
3	*BRCA1*	56	87.5	0	2	0	175	0	Fimbrial
4	*BRCA1*	56	87.5	87.5	2	1	175	87.5	Fimbrial
5	*BRCA1*	50	87.5	87.5	2	1	175	87.5	Fimbrial
6	*BRCA1*	50	87.5	62.5	2	1	175	62.5	Fimbrial
7	*BRCA1*	58	87.5	62.5	2	1	175	62.5	Fimbrial
8	*BRCA1*	52	87.5	87.5	3	2	262.5	175	Fimbrial
9	*BRCA1*	52	87.5	87.5	3	2	262.5	175	Fimbrial
10	*BRCA1*	68	87.5	87.5	1	2	87.5	175	Proximal
11	*BRCA1*	72	87.5	12.5	1	0	87.5	0	Fimbrial
12	*BRCA1*	40	62.5	62.5	0	0	0	0	No difference
13	*BRCA1*	40	12.5	62.5	0	1	0	62.5	Proximal
14	*BRCA1*	48	62.5	62.5	2	2	125	125	No difference
15	*BRCA1*	48	87.5	62.5	3	1	262.5	62.5	Fimbrial
16	*BRCA1*	65	87.5	12.5	2	0	175	0	Fimbrial
17	*BRCA1*	65	87.5	0	2	0	175	0	Fimbrial
18	*BRCA1*	34	87.5	0	1	0	87.5	0	Fimbrial
19	*BRCA1*	53	87.5	62.5	2	1	175	62.5	Fimbrial
20	*BRCA1*	53	87.5	62.5	2	1	175	62.5	Fimbrial
21	*BRCA1*	53	87.5	87.5	2	1	175	87.5	Fimbrial
22	*BRCA1*	72	87.5	87.5	2	1	175	87.5	Fimbrial
23	*BRCA1*	78	87.5	87.5	2	1	175	87.5	Fimbrial
24	*BRCA1*	78	87.5	62.5	1	1	87.5	62.5	Fimbrial
25	*BRCA1*	69	87.5	87.5	3	1	262.5	87.5	Fimbrial
26	*BRCA1*	69	87.5	62.5	2	1	175	62.5	Fimbrial
27	*BRCA1*	43	87.5	62.5	2	1	175	62.5	Fimbrial
28	*BRCA1*	43	87.5	62.5	2	1	175	62.5	Fimbrial
29	*BRCA1*	42	87.5	62.5	2	1	175	62.5	Fimbrial
30	*BRCA1*	42	87.5	87.5	1	1	87.5	87.5	No difference
31	*BRCA1*	53	87.5	87.5	1	2	87.5	175	Proximal
32	*BRCA1*	53	87.5	87.5	2	1	175	87.5	Fimbrial
33	*BRCA1*	48	87.5	87.5	2	1	175	87.5	Fimbrial
34	*BRCA1*	48	87.5	87.5	1	0	87.5	0	Fimbrial

35	*BRCA1*	41	87.5	87.5	2	0	175	0	Fimbrial
36	*BRCA1*	47	87.5	87.5	2	1	175	87.5	Fimbrial
37	*BRCA1*	47	87.5	87.5	2	2	175	175	No difference
38	Control	35	12.5	12.5	0	0	0	0	No difference
39	Control	40	62.5	62.5	1	1	62.5	62.5	No difference
40	Control	49	87.5	87.5	2	1	175	87.5	Fimbrial
41	Control	51	87.5	87.5	2	1	175	87.5	Fimbrial
42	Control	51	87.5	87.5	2	1	175	87.5	Fimbrial
43	Control	49	12.5	12.5	0	0	0	0	No difference
44	Control	52	87.5	87.5	2	1	175	87.5	Fimbrial
45	Control	53	87.5	87.5	1	0	87.5	0	Fimbrial
46	Control	56	62.5	12.5	1	1	62.5	12.5	Fimbrial
47	Control	56	0	0	0	0	0	0	No difference
48	Control	59	87.5	87.5	2	0	175	0	Fimbrial
49	Control	66	87.5	87.5	2	0	175	0	Fimbrial
50	Control	66	62.5	62.5	2	1	125	62.5	Fimbrial
51	Control	69	62.5	62.5	0	0	0	0	No difference
52	Control	69	62.5	62.5	1	1	62.5	62.5	No difference
53	Control	72	62.5	62.5	0	1	0	62.5	Proximal
54	Control	72	87.5	87.5	0	0	0	0	No difference
55	Control	72	87.5	87.5	0	0	0	0	No difference
56	Control	72	87.5	87.5	0	0	0	0	No difference
57	Control	72	87.5	87.5	0	0	0	0	No difference
58	Control	79	87.5	87.5	1	0	87.5	0	Fimbrial
59	Control	70	0	0	0	0	0	0	No difference
60	Control	51	12.5	12.5	1	0	12.5	0	Fimbrial
61	Control	39	12.5	0	1	0	12.5	0	Fimbrial
62	Control	55	62.5	37.5	1	0	62.5	0	Fimbrial
63	Control	51	37.5	12.5	2	1	75	12.5	Fimbrial
64	Control	52	12.5	12.5	1	0	12.5	0	Fimbrial
65	Control	52	12.5	12.5	1	0	12.5	0	Fimbrial
66	Control	69	87.5	87.5	1	1	87.5	87.5	No difference
67	Control	74	87.5	12.5	1	0	87.5	0	Fimbrial
68	Control	74	87.5	12.5	1	0	87.5	0	Fimbrial
69	Control	32	87.5	87.5	1	0	87.5	0	Fimbrial
70	Control	32	0	0	0	0	0	0	No difference
71	Control	68	12.5	0	1	0	12.5	0	Fimbrial
72	Control	55	0	0	0	0	0	0	No difference
73	Control	59	87.5	87.5	1	1	87.5	87.5	No difference

Table S2: Statistical Analysis. *p*-values were calculated using the GraphPad (version 9.5.0) analysis software to perform two-tailed Student t-testing with $p < 0.05$ considered statistically significant. ++paired t-test, +un-paired t-test.

Table S2a: EL phase *BRCA1*/2wt vs. *BRCA1*mut+ (n = 5) as shown in Figure 1b

Time point	*p*-value
12-hours	0.567653
24-hours	0.439103
36-hours	0.457988
48-hours	0.552494
60-hours	0.567374

Table S2b: EF phase *BRCA1*/2wt vs. *BRCA1*mut+ (n = 7) as shown in Figure 1c

Time point	*p*-value
12-hours	0.0371
24-hours	0.0225
36-hours	0.0321
48-hours	0.0873
60-hours	0.1670

Table S2c: P4 dose-dependent effect++ (n = 13) as shown in Figure 1f

Concentration	*p*-value
0.1μM vs. 1.0μM	0.2205
0.1μM vs. 5.0μM	0.0306
0.1μM vs. 10.0μM	0.0038
1.0μM vs. 5.0μM	0.0723
1.0μM vs. 10.0μM	0.0080
5.0μM vs 10.0μM	0.0290

Table S2d: RU-486 (1.25μM) mediated effect++ (n = 15) as shown in Figure 1g

Concentration	*p*-value
10.0μM P4 vs. 10.0μM + RU-486 (1.25μM)	0.0076

Table S2e: Cytoplasmic HIF-1α levels in Fallopian Tube specimens[++] (n = 37) as shown in Figure 2b

**HIF-1α scores in Proximal vs. Fimbrial ends**	***p*-value**
BRCA1/2wt	$p < 0.001$
*BRCA1*mut	$p < 0.0001$

Table S2f: Hypoxic-induced change in NK cell lysis (log transformed *p*-values) (n = 5) as shown in Figure 2d

Time point	*BRCA1*/2wt	*BRCA1*mut
12-hours	$p = 0.1791$	$p = 0.0348$
24-hours	$p = 0.0810$	$p = 0.0213$
36-hours	$p = 0.0400$	$p = 0.0014$
48-hours	$p = 0.0602$	$p = 0.0010$
60-hours	$p = 0.1973$	$p = 0.0039$
