# Supplementary figures and images for: Natural Killer Cell Dysfunction in Premenopausal BRCA1 Mutation Carriers: A Potential Mechanism for Ovarian Carcinogenesis
Source: Cancers (Basel). 2024 Mar 18;16(6):1186. doi: 10.3390/cancers16061186 (PMC10968968; doi:10.3390/cancers16061186)

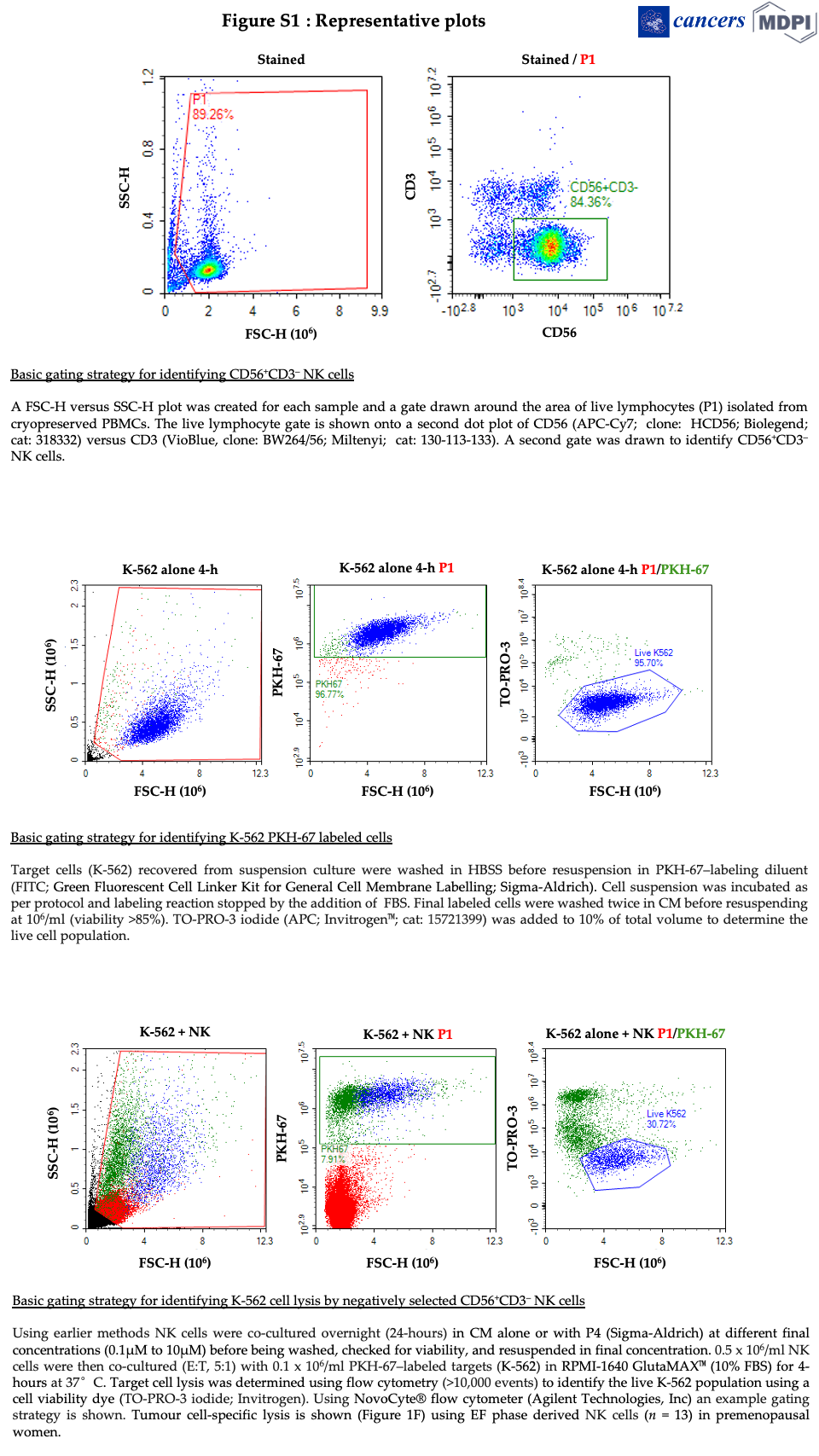

Supplement: Supplementary file 1 [file cancers-16-01186-s001.zip › Figure S1. Representative plots.png]
